# Autoimmune Encephalitis following Checkpoint Inhibitor Therapy in a Patient with Metastatic Melanoma in Complete Remission

**DOI:** 10.3390/medicina60050728

**Published:** 2024-04-27

**Authors:** Giuseppe Civardi, Alessia Medioli, Carlotta Braghieri, Massimo Ambroggi, Paolo Immovilli, Simone Orsucci, Paolo Contini, Giuseppe Aronica, Luigi Cavanna

**Affiliations:** 1 Rehabilitation Unit, Casa di Cura S. Antonino, 29121 Piacenza, Italy; giuseppe.civardi@casadicura.pc.it (G.C.); alessia.medioli@casadicura.pc.it (A.M.); simone.orsucci@casadicura.pc.it (S.O.); continipaolo@gmail.com (P.C.); giuseppe.aronica@casadicura.pc.it (G.A.); 2 Internal Medicine and Oncology, Casa di Cura Piacenza, 29121 Piacenza, Italy; carlotta.braghieri@casadicura.pc.it; 3 Oncology Unit, City Hospital, 29121 Piacenza, Italy; m.ambroggi@ausl.pc.it; 4 Neurology Unit, City Hospital, 29121 Piacenza, Italy; paolo.immovilli.md@gamil.com

**Keywords:** autoimmune encephalitis, checkpoint inhibitor, melanoma, pembrolizumab, adverse event

## Abstract

The use of immune checkpoint inhibitors (ICIs) in cancer is increasing. Their side effects are mainly due to the triggering of autoimmunity, which are mild or moderate and include skin rash, colitis, hepatitis, endocrine disorders, myositis, interstitial lung disorder, etc., in most cases during the course of therapy. Autoimmune encephalitis (AE) is rare in cancer patients treated with ICIs. Fifty patients with ICI-related encephalitis were identified in a recent review. Herein, we report a case of pembrolizumab associated with AE with a favorable short-term prognosis. A 68-year-old man with malignant metastatic melanoma achieved complete remission after pembrolizumab treatment. However, 10 months after pembrolizumab cessation due to grade 3 diarrhea, he developed confusion, an altered mental status, progressive memory loss, and gait disturbance. He was admitted to the neurologic department, and a comprehensive neurological workup, brain magnetic resonance imaging, cerebral fluid analysis, EEG, and blood test allowed the diagnosis of autoimmune encephalitis. The patient was treated with plasmapheresis, a high dose of intravenous steroids, and intravenous immunoglobulins. The patient improved, and he is now well with a performance status of 1. This case is interesting since the AE developed approximately 10 months after the cessation of immunotherapy, the underlying cancer was in complete remission, and the AE showed a good response after the treatment was performed.

## 1. Introduction

Immunotherapy has become an important clinical strategy in the treatment of cancer patients.

Immune checkpoint inhibitors (ICIs) are monoclonal antibodies that enhance anti-tumor immune activity by activating T-cells [1,2].

The anti-tumor effects of ICIs have been demonstrated in several randomized clinical trials, and ICIs are now available for the treatment of many malignant cancers, such as lung cancer, melanoma, hepatocellular carcinoma, and gastrointestinal cancer.

Immune-related adverse events (IRAEs) may be associated with ICIs and may occur at any time after the initiation of ICI treatment [3].

Most IRAEs are mild and moderate and include skin rash, colitis, hepatitis, endocrine disorders, myositis, and interstitial lung disorder [3]. IRAEs involving the nervous system are relatively uncommon and include myasthenia gravis, Guillain-Barre syndrome, and peripheral sensory-motor neuropathy [4].

ICI-associated autoimmune encephalitis is infrequent, and this complication is more common during concurrent or sequential ICI treatment and in patients with lung cancer [5,6]. Fifty patients with ICI-related autoimmune encephalitis were identified in a review of cases published from 2016 to 2022 [4].

Herein, we report a case of autoimmune encephalitis in a patient with metastatic melanoma in complete remission after pembrolizumab treatment.

## 2. Case Report

**Figure 1:**
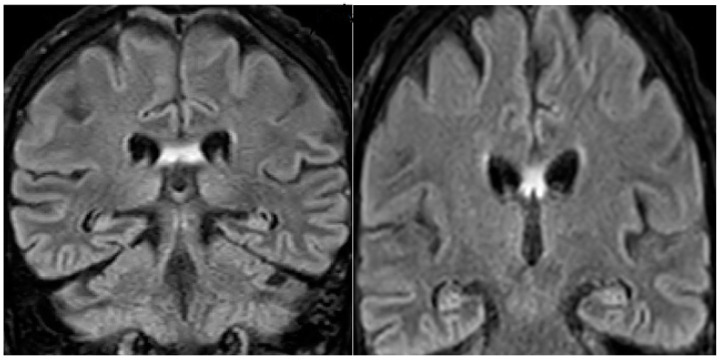
Brain MRI of the patient showing bilateral fornix hyperintensity in the FLAIR (fluid-attenuated inversion recovery) sequences.

A 68-year-old man was referred to the neurologic department hospital of Piacenza (North Italy) in December 2023 with approximately a 3-month history of worsening gait, weakness, loss of appetite, and a confusional state. The patient was diagnosed with malignant melanoma in his left hand in April 2018. Primary melanomas of the third finger, last phalanx, and left hand were diagnosed, and the patient underwent amputation of the phalanx. A histological examination showed T4b stage IIC ulcerated melanoma. The mutation status was negative for the BRAF V600E mutation, and the patient underwent a complete staging with total body computerized tomography (CT) scans, which were negative for metastasis. The patient, 3 years later, developed lung and liver metastases, one metastasis of 2 cm in diameter at the left liver lobe and one metastasis at the superior left lung lobe of 1.5 cm in diameter. Treatment with pembrolizumab 200 mg every 3 weeks was then initiated on 15 July 2021. After six months of pembrolizumab, restaging with CT and FDG-PET/CT showed complete remission. The treatment was continued for 14 months and then stopped due to grade 3 diarrhea. The patient was in complete remission when, 10 months after the cessation of pembrolizumab therapy, he developed the following neurological symptoms: confusion, an altered mental state, progressive memory loss, and gait disturbance. The neurological examination did not display focal deficits. Cognitive testing revealed MMS 18/30. Head magnetic resonance imaging did not reveal brain metastasis, signs of carcinomatous meningitis or stroke, and evidenced hyperintensity in the fornix bilaterally on flair imaging (Figure 1).

The EEG showed slower asymmetric activity in the right cerebral hemisphere. The cerebrospinal fluid (CSF) examination showed signs of inflammation, with a raised protein and lymphocyte count but no malignant cells. The viral PCR was negative. Anti-SOX1 antibodies were detected in the serum and CSF. The total body CT and PET/CT were negative for the relapse of melanoma or other malignancies.

Autoimmune encephalitis was suspected as the patient was previously treated with pembrolizumab, and he did not fulfill the criteria for definite or possible paraneoplastic neurological syndrome since no evidence of malignant disease was found with the total body CT and PET/CT. Furthermore, the clinical/laboratory findings were coherent with the recently published Canadian consensus guidelines for the diagnosis and treatment of autoimmune encephalitis in adults [7]. The patient was then treated with two plasmapheresis sessions, then high doses of steroids (methylprednisolone 1 g daily via IV for 5 days followed by prednisone 62.5 mg daily orally), intravenous immunoglobulins at 0.4 g/kg/day IV for 5 days. The neurological symptoms improved significantly, and he underwent a physical rehabilitation physiotherapy program at the St. Antonino clinic (Piacenza).

The patient was discharged from the St. Antonino clinic on 20 January 2024. He is now well, showing an improved neurological status, and is now under observation for melanoma in complete remission and for autoimmune encephalitis, with progressive improvements in neurologic symptoms and a performance status of 1. The patient was reviewed and deemed recovered on 23 April 2024, with a performance status of 0.

## 3. Discussion

Immune checkpoint inhibitors have demonstrated important results in many types of cancer. Inhibitors of cytotoxic T-lymphocyte-associated antigen 4 (CTLA-4), programmed death-1 (PD-1), and programmed death receptor ligand 1 (PD-L1) are currently being used in patients with cancer [1,2,8]. In these treatments, the primary mechanism of action is the generation of an immune response against cancer rather than performing actions directly at the tumor [8]. This mechanism can allow complete tumor regression associated with an improved quality of life compared with cytotoxic chemotherapy and or radiation.

It must be remembered that the 2018 Nobel Prize in Medicine and Physiology was awarded to James Allison and Tasuku Honjo for their studies and discoveries in cancer immunology-based treatment [9].

James Allison discovered the immunosuppressive molecule cytotoxic T-lymphocyte antigen 4, and Tasuku Honjo discovered the programmed death molecule-1 on T-cells.

The major escape mechanism of cancer cells is the suppression of T-cell activation by CTLA-4 or by PD-1. Immune checkpoint inhibitors promote the inhibition of these molecules and, consequently, the activation of the immune system against cancer cells.

ICIs offered a new and effective treatment against cancer; effectively, before the advent of ICIs, the therapeutic standard for treating cancer was based on surgery, chemotherapy, radiation therapy, and, recently, target therapy [9]. Ipilimumab was the first immune checkpoint inhibitor available against cancer; this anti-CTLA-4 antibody was able to produce a durable response and survival in patients with metastatic melanoma [10]. ICIs are monoclonal antibodies that act by blocking the inhibitory molecules involved in the regulation of immune response pathways, such as programmed cell death-1 (e.g., cemiplimab, nivolumab, and pembrolizumab) or programmed cell death protein ligand 1 (e.g., atezolizumab, avelumab, and durvalumab) or cytotoxic T-lymphocyte-associated protein 4 (e.g., ipilimumab). It must be emphasized that immune checkpoint inhibitors, by activating T-cells, can trigger a large spectrum of autoimmune responses that are currently referred to as immune-related adverse events [11]. These adverse events deeply differ from the toxicities caused by cytotoxic chemotherapy or by molecularly targeted drugs; it must be kept in mind that the time to toxicity may be delayed and not predictable, as seen with conventional anti-cancer chemotherapy or radiation therapy [11]. The incidence and pattern of IRAEs are different according to the type of ICIs used: hypophysitis and colitis were more frequent with ipilimumab, while diabetes and pneumonia were more frequent with anti-PD1/PD-L1 in a large review that analyzed 16,485 patients treated with ICIs in randomized clinical trials [12].

However, real-world data indicate that the incidence of ICI-related IRAEs may be higher than previously highlighted in clinical trials [13]. The rates of toxicity are higher with CTLA-4 inhibitors when compared with PD1/PD-L1 inhibitors, and IRAEs can affect any organ system [13]. The risk factors for IRAEs are not well understood; however, there is evidence suggesting that IRAEs are more frequent and occur faster in patients with pre-existing autoimmune diseases, such as psoriasis, rheumatoid arthritis, vasculitis, intestinal bowel disease, systemic lupus erythematosus, and patients with underlying autoimmune diseases should be managed by a multidisciplinary team [13]. The combination therapy of anti-CTLA-4 and anti-PD1/PD-L1 favors a greater incidence of high-grade IRAEs [13].

The management of IRAEs is predominantly based upon retrospective studies, and glucocorticoids represent the first-line therapy option for most ICI-induced adverse events, though glucocorticoids may be associated with a reduction in the anti-tumor effect of ICIs. Low doses of steroids do not seem to affect the tumor response and the survival of cancer patients treated with ICIs.

For patients with IRAEs that are not responsive to glucocorticoids, treatment with monoclonal antibodies targeting TNF-α, such as infliximab, was effective in autoimmune colitis and pneumonia during ICI therapy [13].

The timing of ICI toxicity should be considered with caution since IRAEs can occur late during treatment with ICIs or after treatment discontinuation [13], as reported in our case.

This case describes pembrolizumab-associated autoimmune encephalitis in a 68-year-old male with metastatic melanoma in complete remission. It is very interesting that autoimmune encephalitis was diagnosed 10 months after pembrolizumab therapy cessation.

In our patient, the presence of the anti-SOX1 antibody was detected in both the serum and CSF, as reported in previous cases of immune checkpoint inhibitors associated with autoimmune encephalitis [4].

In adults, anti-SOX1 antibodies are classified as onconeural antibodies and were first reported in patients with Lambert-Eaton myasthenic syndrome (LEMS) and small cell lung cancer (SCLC); they were considered a marker of SCLC-related paraneoplastic syndrome [14]. The main clinical manifestations of LEMS are predominantly characterized by proximal muscle weakness and absent or very reduced tendon reflexes. Other symptoms include constipation, dry mouth, and erectile dysfunction [14].

Symptoms of LEMS can precede months or even years before the diagnosis of small cell lung cancer. In recent years, the detection of LEMS-related antibodies has facilitated the diagnosis of this rare autoimmune neuromuscular junction disorder, and a series of reports have demonstrated that the SOX1 antibody, initially called the anti-glial nuclear antibody (AGNA), is associated with Lambert-Eaton myasthenic syndrome [14,15]. However, subsequently, anti-SOX1 antibodies were also detected in patients with various neurological disorders, predominantly immune-mediated diseases [15], as in patients with ICI-associated autoimmune encephalitis [4].

Recently, SOX1 antibodies were detected in a 3-year-old girl who presented with ataxia and dysmetria; she had a recent varicella infection. The tests performed were the magnetic resonance imaging of the brain and spinal cord, an EEG, blood and urine tests, and a lumbar puncture, which did not show signs of encephalitis; only anti-SOX1 antibodies were identified in both the serum and CSF. The patient showed a favorable clinical course, with rapid improvements in the symptoms. This case report shows that SOX1 antibodies may also be found in childhood after viral infections like varicella [16]. Within these cases of ICI-related encephalitis, Gao Y. et al. [4], reviewing English literature in the PubMed database published in 2022, found 50 cases of ICI-associated autoimmune encephalitis; the majority of these cases (20/50) had lung cancer (40%). Interestingly, the time from the beginning of immune checkpoint inhibitor therapy to autoimmune symptom onset ranged from 4 days to 18 months (median: 3 months). In this review of the literature, the treatments performed for autoimmune encephalitis were steroids, intravenous immunoglobulins, plasmapheresis, and rituximab. Thirty-one patients out of fifty (62%) improved, thirteen patients (26%) did not improve, and six patients (12%) died. Our patient was treated with plasma exchange, high doses of steroids, and intravenous immunoglobulins with a slow but progressive improvement. More recently, Fonesca E. et al. [6] reported neurological adverse events related to immune checkpoint inhibitors in a retrospective cohort study, which included 64 patients in Spain. Forty-five out of sixty-four (70.3%) patients showed encephalopathy. In these patients, neurological symptoms developed very soon (median: 8 weeks) after treatment with immune checkpoint inhibitors compared to our case, in which neurological symptoms developed 10 months after the cessation of the treatment with pembrolizumab.

In patients reported by Fonesca et al. [6], twelve out of forty-five patients with encephalopathy (27%) had definite paraneoplastic or autoimmune encephalitis, twenty-four (53%) had encephalitis without antibodies, and nine (20%) showed encephalopathy without central nervous system (CNS) inflammation changes. Around 40% of the entire group of patients died during the study period, and in 12 of the 27 patients who died, the cause of death was a neurological immune-related adverse event. Interestingly, most of the deaths occurred during the first month after the onset of symptoms related to adverse events. The mortality risk was associated with lung cancer when compared with other cancers and in patients with encephalopathy without evidence of CNS inflammation or combined myocarditis, myasthenia, and myositis [6].

Farina et al. [17] performed a large multicenter retrospective cohort study of patients in France in order to assess the neurological adverse events associated with ICIs, and in this series of 147 cases, 51 patients (34.7%) with CNS involvement were described, while in 87/147 patients (59.2%), the peripheral nervous system was involved. In all 51 patients with CNS involvement by IRAEs, the cerebrospinal fluid examination excluded the presence of malignant cells; an MRI of the brain excluded the presence of CNS metastasis. Anti-neuronal/glial antibodies were detected in 64.4% of the tested patients, and SOX1 antibodies were detected in three patients. ICIs were discontinued due to neurologic adverse events in 99.5% of the patients; the median delay from the first ICI dose to the last ICI dose was 40 days (range: 0–760). In 51.1% of the patients that withdrew from ICI treatment, cancer progression was documented, 11.5% of patients were rechallenged with ICIs, and 2/17 (11.7%) showed a neurologic relapse. Neurologic adverse events were treated with steroids in 93% of the patients, intravenous immunoglobulin was administered in 36.8% of the patients, and plasma exchange was administered in 15.3% of the patients. The mortality rates increased gradually at 6, 12, and 18 months of follow-up, and 32.7% of the patients died during follow-up. The main causes of death were cancer progression in 35.4% of patients, neurological toxicity (31.2%), and other causes (20.8%). Interestingly, the most frequent cause of death was neurological toxicity in the first 3 months after the onset of neurological adverse events (64.3%), while cancer progression became the most predominant cause after 3 months (47.1%).

In this study, neurological recovery after IRAEs was possible even in cases with severe presentations, in patients with melanoma, and in cases with myositis/neuromuscular junction disorders. In these series, older age and paraneoplastic-like syndromes showed poor neurological outcomes.

In a retrospective study at the Mayo clinic, 16 patients with encephalitis related to ICIs were reported. The majority of these patients received immunosuppressive treatment, and ICIs were discontinued in 97% of the patients. The unfavorable outcome was described in 39% of the entire reported series, and it was associated with a higher severity degree at onset and a shorter period from ICIs to the onset of neurological symptoms [18].

## 4. Conclusions

In conclusion, the use of ICIs alone or in combination is increasing for multiple tumor types. ICIs-related encephalitis, though rare, exists, and it should be suspected in patients treated with ICIs presenting neurological symptoms like confusion, gait disturbance, a reduced level of consciousness, and progressive memory loss. In these patients, neurological workups, blood tests, and cerebral MRI, EEG, and CSF analyses should be performed. The treatments should be based on high-dose intravenous steroids, intravenous Ig, and plasma exchange. The second-line treatment should be based on rituximab and cyclophosphamide infusion. Early involvement of a specialist in autoimmune neurology is recommended in patients undergoing consideration for second-line therapy [7].

However, we recall to the mind that ICIs have revolutionized the treatment of many types of cancer, and many patients with cancer treated with ICIs present a significant improvement in survival compared with previous standards of anti-tumor therapy [19].

## Data Availability

The data presented in this study are available upon request from the corresponding author.
